# Acute Posterior Multifocal Placoid Pigment Epitheliopathy Associated with Gastrointestinal Stromal Tumor and Hurthle Cell Tumor

**DOI:** 10.1155/2018/1656131

**Published:** 2018-09-30

**Authors:** Daniel D. Kim, Ghassan Ghorayeb

**Affiliations:** Eye Institute, West Virginia University, Morgantown, WV, USA

## Abstract

Acute posterior multifocal placoid pigment epitheliopathy (APMPEE) is a chorioretinal inflammatory disease of unknown origin. Patients usually present with a rapid loss of central/paracentral vision over the course of a week in both eyes. The fundus exhibits rapid appearance of multiple deep subretinal yellow-white, flat lesions at the RPE/choriocapillaris level. This in turn causes changes of both the ellipsoid zone and RPE which can result in permanent central vision loss. The pathogenesis is controversial but is associated with a recent viral illness and can involve the central nervous system with concern for cerebral vasculitis. Rare reports of APMPEE associated with systemic vasculitis such as Wegener's granulomatosis and malignancy such as clear cell renal carcinoma have been reported. We report a case of APMPEE with concurrent diagnosis of gastrointestinal stromal tumor and Hurthle cell tumor. While such association may well be coincidental, the near simultaneous presentation raised our suspicion for potential association.

## 1. Background

Acute posterior multifocal placoid pigment epitheliopathy (APMPEE) is a chorioretinal inflammatory disease of unknown origin. The primary tissue involved is thought to be the retinal pigment epithelium (RPE) and/or the choriocapillaris [1].

Patients usually present with a rapid loss of central/paracentral vision. Typically, over the course of a week, both eyes become involved with vision loss but can be asymmetrical [2]. Visual recovery is generally rapid and of good visual acuity but can leave varying degrees of persistent metamorphopsia and scotomas [3].

The fundus exhibits rapid appearance of multiple deep subretinal yellow-white, flat lesions at the RPE/choriocapillaris level. Parafoveal lesions of varied sizes are more common but involve the macula and early periphery. The lesions fade over weeks and leave hypo/hyperpigmentation as well as RPE atrophy. Fluorescein angiogram (FA) features early hypofluorescence followed by late staining of the lesion. Indocyanine green angiography shows early and late hypofluorescence. Optical coherence tomography (OCT) shows early in the disease heterogenous subretinal fluid which resolves and outer nuclear layer hyperreflectivity which resolves into thinning. Concurrent disruption of the ellipsoid zone with hyperreflectivity of the RPE can persist for months [4].

The pathogenesis is controversial but is associated with a recent immune response and can involve the central nervous system ranging from headaches to rarely reported cerebral vasculitis [5, 6]. There have also been case reports with association of viruses [7, 8], Wegener's granulomatosis [9], and renal cell carcinoma [10]. We report a case of APMPEE wherein the patient was also diagnosed with two different tumors: a gastrointestinal stromal tumor (GIST) and a Hurthle cell tumor.

## 2. Case Presentation

A 50-year-old Caucasian male presented with 5 days of significant central vision changes in both eyes. About 4 days prior to visual symptoms, he started a viral like illness with severe headaches, fevers, chills, and joint pain. He was given Tamiflu by an urgent care clinic after being diagnosed with the flu.

**Figure 1:**
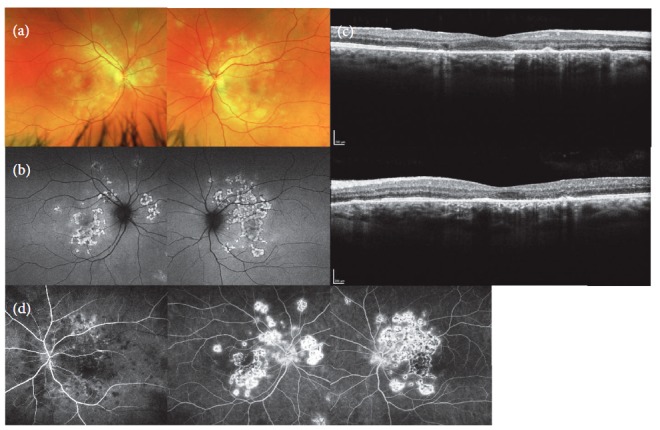
Images taken 3 days after onset of symptoms. Fundus photo, yellowish white subretinal placoid lesions (a). FAF, placoid lesions with central hypoautofluorescence with hyperautofluorescent edges (b). OCT Fovea, top: OD and bottom: OS. RPE and ellipsoid zone attenuation of placoid lesions. Subretinal hyperreflective material (c). FA, left: early phase OS. Central and right: late phase OD and OS. Early blocking with late staining (d).

His vision was found to have a best corrected visual acuity of (BCVA) 20/25 OD and count fingers OS. Intraocular pressures: 14mmHg OD 15mmHg OS. Brisk pupil reactions were found with no afferent pupillary defect in both eyes. Extraocular movements were full. Anterior chamber examination showed normal cornea, iris, and lens with deep chambers and no cell/flare in both eyes. Posterior segment examination showed a clear media with no vitritis as well as normal disc and vessels. There were, in the posterior pole of both eyes, multiple yellow-white chorioretinal placoid lesions more significant on the left eye (Figure 1(a)).

Spectral domain optical coherence tomography showed the placoid lesions with disruption of the RPE, external limiting membrane, and ellipsoid zone as well as small focal points of hyperreflective material at the level of the ellipsoid zone (Figure 1(c)). Fundus autofluorescence (FAF) showed the placoid lesions to have hyperautofluorescence center with hypoautofluorescence edges (Figure 1(b)). Fluorescein angiogram showed the placoid lesions had the characteristic early blocking with late hyperfluorescent staining of edges (Figure 1(d)). Based on imaging and clinical exam, the patient was diagnosed APMPEE.

Due to the concern for cerebral vasculitis, the patient was admitted for imaging and treatment. A lab work-up showed an elevated ESR and CRP, positive IgG toxocara, and toxoplasma. IgM toxocara and toxoplasma were negative and the rest of the lab workup was negative. MRI brain, CTA head/neck, and lumbar puncture performed were found to be normal. After ruling out infectious causes, the patient was started on intravenous high dose steroids with transition to PO steroids of 1mg/kg and a planned slow taper.

After a couple of weeks of starting steroids, the patient had an incidence of bright red blood per rectum and underwent a rapid steroid taper as well as a colonoscopy. A biopsy was performed during the colonoscopy which showed a gastrointestinal stromal tumor. The lesion later excised showed on pathologic analysis a high grade gastrointestinal stromal tumor. The patient was advised that he may need adjuvant chemotherapy.

Also during work-up of the GIST, a thyroid nodule was found. Subsequent fine needle biopsy of the lesion showed atypical Hurthle cells. Genetic testing of the atypical cells showed benign characteristics with low malignant potential. The patient currently is pending excision of the thyroid lesion.

**Figure 2:**
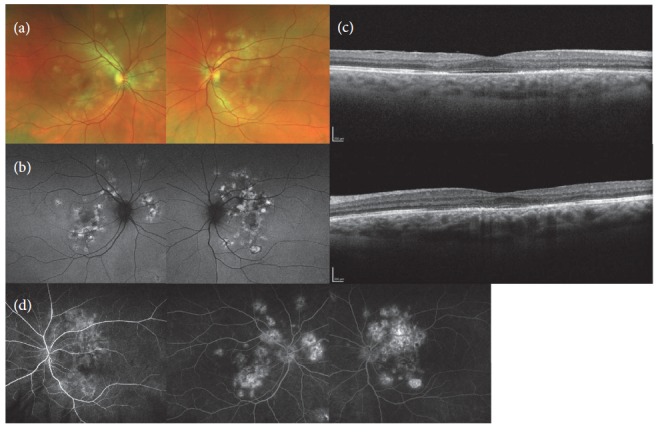
Images taken 3 months after onset of symptoms. Fundus photo, yellowish white subretinal placoid lesions smaller in size (a). FAF, placoid lesions with central hypoautofluorescence with hyperautofluorescent edges, smaller in size and boundaries less distinct (b). OCT Fovea, top: OD and bottom: OS. Much improved RPE and ellipsoid zone attenuation of placoid lesions and resolved subretinal hyperreflective material (c). FA, left: early phase OS. Central and right: late phase OD and OS. Early blocking with late staining less than before (d).

On the 3-month follow-up visit on no systemic steroids, the BCVA stabilized at 20/25 with the patient's paracentral scotomas persisting. Imaging showed maturing of the lesion with stable size on FAF, more apparent late staining on FA, and mild improvement of the ellipsoid zone on the edges of the lesions on OCT (Figures 2(b), 2(c), and 2(d)). On the 5-month follow-up, the BCVA was 20/20 OU.

## 3. Discussion

To our knowledge this is first case of APMPEE diagnosed along two different tumors: a gastrointestinal stromal tumor and a Hurthle cell tumor. Further investigation will be needed over time to see if the two tumors are associated with APMPEE.

There are a couple of known associations with APMPEE: cerebral vasculitis and viral illness. Both associations have been well described in the literature. Case et al. note a review of literature of 23 case reports with ischemic and/or hemorrhagic stroke secondary to cerebral vasculitis [5, 6]. Viral illness also has been described [7]. More rarely Wegener's granulomatosis [9] and other infectious causes such as borreliosis [11] have been linked to APMPEE.

There is also a case report of a renal cell carcinoma associated with APMPEE. It was speculated that circulating immune complexes from the renal cell carcinoma may have been the cause of APMPEE [10]. For our patient, both solid tumors are not known to secrete circulating factors or proteins. The tumors do have specific genetic components that affect treatments such as imatinib for GIST [12]. We certainly acknowledge that this association may be coincidental, yet the near simultaneous presentation raises the suspicion as to if and how these tumors are related to APMPEE.

This case, much like other APMPEE diagnoses, has a history of viral prodrome, but both tumors were present and certainly affected the patient's immunological state. This unique case gives incentive to consider other causes or associations including neoplasm for APMPEE.
